# Accelerated Variant of Idiopathic Pulmonary Fibrosis: Clinical Behavior and Gene Expression Pattern

**DOI:** 10.1371/journal.pone.0000482

**Published:** 2007-05-30

**Authors:** Moisés Selman, Guillermo Carrillo, Andrea Estrada, Mayra Mejia, Carina Becerril, José Cisneros, Miguel Gaxiola, Rogelio Pérez-Padilla, Carmen Navarro, Thomas Richards, James Dauber, Talmadge E. King, Annie Pardo, Naftali Kaminski

**Affiliations:** 1 Instituto Nacional de Enfermedades Respiratorias, Mexico City, Mexico; 2 The Dorothy P. and Richard P. Simmons Center for Interstitial Lung Diseases, Pulmonary Allergy and Critical Care Medicine, University of Pittsburgh, Pittsburgh, Pennsylvania, United States of America; 3 Department of Medicine, Division of Pulmonary and Critical Care Medicine, San Francisco General Hospital, San Francisco, California, United States of America; 4 Facultad de Ciencias, Universidad Nacional Autónoma de México, Mexico City, Mexico; Washington University, United States of America

## Abstract

**Background:**

Idiopathic pulmonary fibrosis (IPF) is characterized by the insidious onset of dyspnea or cough. However, a subset of patients has a short duration of symptoms with rapid progression to end-stage disease. In this study, we evaluated clinical and molecular features of “rapid” and “slow” progressors with IPF.

**Methods and Findings:**

26 patients with <6 months of symptoms before first presentation [rapid progressors] and 88 patients with >24 months of symptoms [slow progressors] were studied. Survival was analyzed by the Kaplan-Meyer method and proportional hazard's model. Lung microarrays and tissue proteins were measured in a subset of patients. No differences were found in age, physiologic impairment and bronchoalveolar lavage (BAL) cellular profile. There were more males (OR = 6.5; CI:1.4-29.5; p = 0.006) and smokers (OR = 3.04; CI:1.1-8.3; p = 0.04) in the rapid progressors group. Survival from the beginning of symptoms was significantly reduced in rapid progressors (HR = 9.0; CI:4.48-18.3; p<0.0001) and there was a tendency for decreased survival from the time of diagnosis (HR = 1.5; CI:0.81-2.87; p = 0.18). We identified 437 differentially expressed genes. Lungs of rapid progressors overexpressed genes involved in morphogenesis, oxidative stress, migration/proliferation, and genes from fibroblasts/smooth muscle cells. Upregulation of two of these genes, adenosine-2B receptor and prominin-1/CD133, was validated by immunohistochemistry and were expressed by alveolar epithelial cells. BAL from rapid progressors showed a >2-fold increase of active matrix metalloproteinase-9, and induced a higher fibroblast migration compared with slow progressors and controls [238±98% versus 123±29% (p<0.05) and 30±17% (p<0.01)].

**Conclusions/Significance:**

A subgroup of IPF patients, predominantly smoking males, display an accelerated clinical course and have a gene expression pattern that is different from those with slower progression and longer survival. These findings highlight the variability in the progression of IPF, and may explain, in part, the difficulty in obtaining significant and reproducible results in studies of therapeutic interventions in patients with IPF.

## Introduction

Idiopathic pulmonary fibrosis (IPF) is a chronic fibrosing interstitial lung disease of unknown etiology characterized by progressive dyspnea, reduced lung volumes, impaired gas exchange, and the histopathologic signature of usual interstitial pneumonia (UIP). This disease, which is the most common of the idiopathic interstitial pneumonias, is unresponsive to current therapy and most patients die within 5 years after diagnosis [1]–[3]. However, it is increasingly apparent that IPF patients exhibit distinct patterns of disease progression [4], [5]. Most of them show a long duration of symptoms before diagnosis and then experience a slowly progressive clinical course (“slow” progressors) [5]. Often, an acute clinical deterioration (“acute exacerbation” of IPF) precedes the terminal phase of the illness in this subgroup [5], [6]. Quite distinct from these observations, some IPF patients display a more rapidly progressive clinical course with a shorter duration of symptoms before diagnosis and progression to death (“rapid” progressors). However, a systematic characterization of these distinct disease progression phenotypes has not been performed.

The purpose of this study was to determine whether “rapid” and “slow” progressor IPF patients could be distinguished by clinical, biological or molecular features. Better identification and understanding of these differences may provide insights into the pathogenesis and assist in the development of therapeutic interventions.

## Methods

### Study Population

This study included 114 individuals from a cohort of 167 consecutive patients with IPF evaluated at the National Institute of Respiratory Diseases, Mexico, between 1995 and 2004. The study was approved by the Ethics Committee of the National Institute of Respiratory Diseases, México. Diagnosis of IPF was made based on established criteria and confirmed by lung biopsy in 31% of the subjects [7]. Clinical data (time of symptoms before diagnosis, smoking status, drug treatment, clinical findings, absence of previous environmental exposures, and collagen-vascular disease) were extracted from case records. The duration of illness was defined in two ways: (1) the time from the onset of the disease, determined from the patient's recollection of the first appearance of dyspnea or cough throughout the day; and (2) the time from the clinical diagnosis of IPF.

Smoking status was characterized as “never”, “former” (patients who stopped smoking at least 12 month before presentation), or “current” (patients who were either still smoking or stopped smoking less than a year before presentation) [8]. Smoking index (packs/year) was also documented.

Patients were treated with several regimens: prednisone, or prednisone plus azathioprine, or inhaled beclomethasone. Most patients also received colchicine. There were no differences between the type of initial treatment among both groups (data not shown).

### Control Subjects

Seven healthy volunteers (43.4±11.9 years) were selected as controls for the gelatin zymography study, and five of them were assayed for fibroblast migration. Lungs from patients who died from non-respiratory causes (53.7±6.7 years) and from patients with chronic hypersensitivity pneumonitis (56.1±8.9 years) were used as controls for immunohistochemistry.

### Lung Function and Imaging Studies

Pulmonary function tests, including spirometry, plethysmography, and arterial blood gases were performed as described elsewhere [5], [9], [10]. Separate comparisons of oxygen saturation (SpO_2_) levels were performed at the baseline and after 6 months follow-up using Wilcoxon rank-sum tests. A longitudinal test for group differences at 6 months controlling for baseline oxygen saturation, was performed using analysis of covariance (ANCOVA) to adjust for regression toward the mean.

High resolution computed tomography (HRCT) was performed with 1.0- or 1.5-mm-thick axial sections taken at 1-cm intervals throughout the entire thorax and were reconstructed using a high spatial frequency algorithm. Between 20 and 25 CT images were acquired in each patient. HRCT scans were scored on a scale of 0-5 for ground glass attenuation, extent of septal thickening and honeycombing as described [10]. Also, to determine the effect of smoking on lung architecture, the percent of emphysematous lesions was quantified.

### Bronchoalveolar lavage

As part of the diagnosis process, bronchoalveolar lavage (BAL) was performed in 85 out of the 114 patients as described [11]–[13]. Cells were stained with hematoxylin&eosin for differential cell counts. Supernatants were frozen at −70° until use.

### Histopathologic Evaluation

Tissue samples were obtained by open lung biopsy in 8 from 26 “rapid” and 27 from 88 “slow” progressors. None of the patients had been treated with corticosteroids or immunosuppressive drugs at the time of biopsy. There was no mortality related to the surgical procedure and all patients were discharge from the hospital. One “rapid” progressor patient and two “slow” progressors showed surgical morbidity which included prolonged air leakage (≥6 days, 1 patient in each group) and hemothorax in 1 “slow” progressor patient. Lung samples were fixed with 10% formaldehyde and handled routinely for light microscopy. A pathologist, blinded to the clinical data, scored the lesions from 0–2 (absent, mild/moderate and severe): 1) extent of honeycomb; 2) hyperplasia of smooth muscle cells; 3) hypertensive changes; 4) extent of fibrosis 5) extent of interstitial inflammation; 6) hyperplasia of type 2 cells. The assessment was done as previously described [14], [15]. Hyaline membranes were evaluated as present or absent. Fibroblastic foci (FF) were counted using hematoxylin&eosin and Massońs trichrome. Each biopsy was viewed at low-power magnification (x40), and the numbers of FF were counted within all tissue specimen. The area of the tissue sample was measured and results were expressed as FF/cm^2^.

### Immunohistochemistry

Lung tissue sections from 7 “rapid” progressors 8 “slow” progressors, 5 hypersensitivity pneumonitis, and three control lungs were treated as previously described [12], [13]. Rabbit anti-human adenosine 2B receptor (A_2B_AR) (5 µg/ml) (Chemicon Int, Tamecula CA) and mouse anti-human prominin-1/CD133 (10 µg/ml) (clone AC133; Miltenyi Biotec, Auburn, CA), were applied and samples were incubated at 4°C overnight. A secondary biotinylated anti-immunoglobulin followed by horseradish peroxidase-conjugated streptavidin (BioGenex, San Ramon CA) was used according to manufacturer's instructions. 3-amino-9-ethyl-carbazole (AEC, BioGenex) in acetate buffer containing 0.05% H_2_O_2_ was used as substrate. The sections were counterstained with hematoxylin. The primary antibody was replaced by non-immune serum for negative control slides.

### BAL gelatin zymography

To identify gelatinolytic activity, BAL fluid samples from 8 “rapid” progressors, 8 “slow” progressors and **7** controls (1.5 µg of protein) were analyzed in 8.5% SDS-PAGE gels containing gelatin (1 mg/ml) and a final concentration of 0.3 mg/ml heparin as previously described [16]. Human matrix metalloprotease (MMP)-2 and MMP-9 zymography standards (Chemicon, CA) were used as gelatinolytic markers.

### Western Blot Analysis

BAL fluid samples were 50× concentrated by lyophilization and solubilized in water. Aliquots containing 35 µg of protein were mixed with 2× Laemmli buffer (V/V) and separated on 10% SDS-polyacrylamide gels. Proteins were electroblotted onto nitrocellulose membranes (Hybond ECL, Amersham Biosciences). After blocking with 5% (w/v) non-fat dried milk in PBS, the membranes were incubated overnight at 4°C with polyclonal antibody against human adenosine A2B receptor (2 µg/ml; Chemicon, Tamecula, CA), and monoclonal antibody against CD-133 (1 µg/ml; ABGENT, San Diego, CA). Membranes were subsequently washed, incubated with the corresponding secondary antibody conjugated to horseradish peroxidase (1∶20000; Jackson, West Grove, PA) for 1 h at room temperature, and visualized with Enhanced Chemiluminescence (ECL) detection system (Amersham Biosciences, UK) using radiograph film (Hyperfilm, Amersham Biosciences) according to the instructions of the manufacturer. Films were digitalized and quantified using image analysis software (ID; Eastman Kodak Company; Rochester, NY).

### RNA extraction and DNA microarray hybridization

Lung samples from 4 “rapid” and 4 “slow” progressor patients were among the samples previously described by us [13]. However, the gene expression results presented in this manuscript have not been previously published. RNA extracted from lung tissue was used to generate labeled cRNA and hybridized to a custom Affymetrix oligonucleotide microarray (Hu03 containing 59,619 probesets representing 29655 transcripts) that were scanned and normalized as described [13]. The dataset is available at http://www.dom.pitt.edu/paccm/genomics/ACC/index.htm.

Statistical analyses were performed as described [17], [18] using ScoreGenes software package (http://compbio.cs.huji.ac.il/scoregenes/). For Data Mining and visualization, we used Genomica (http://genomica.weizmann.ac.il/) and Spotfire Decision Site 8.0 (Spotfire Inc. Göteborg, Sweden). Correction for only transcripts that had an Entrez Gene annotation was included in the analysis.

To identify genes that best distinguish between “rapid” and “slow” progressors, we used the Threshold Number of Misclassification (TNoM) score [19] as well as the Student's t-test. TNoM score counts the number of classification errors that occur between compared groups for each gene of the dataset. To improve the stringency of our analysis we considered genes as changed only if they had a t-test and a TNoM p-value <0.05 and a fold ratio >2 as previously described [20].

### Migration assay

Migration of normal human lung fibroblasts was performed using 24-well collagen-coated Boyden chambers (Chemicon Temecula, CA) as described [11]. Fibroblasts were derived from a region of the lung showing no histologic abnormalities of a patient undergoing lobectomy for removal of a solitary pulmonary nodule. Fibroblasts (3×10^5^ cells) were added to the upper chamber. The lower chamber contained 0.3 ml of medium with 5% BSA alone or with 50% BAL fluid. After incubation for 8 h at 37°C the non-migrating cells on the top of the chamber were scraped and washed. The migrating cells were determined according to manufacturer's instructions. Briefly, the cells were stained and the color was eluted with 300 µl of extraction buffer and aliquots of 150 µl were measured in an ELISA plate reader at 545 nm. The number of cells that migrated in absence of BAL was used as control (0% migration). All assays were performed in duplicate.

### Statistical methods

Data were analyzed with STATA software. In August 2005, we obtained the vital status of each patient by reviewing the clinical charts and using telephone or telegrams in all cases that had been lost to follow up. We obtained the vital status on 85% of the “rapid” progressors and of 80% of the “slow” progressors. For the remaining patients, their vital status was considered alive. Data are summarized using mean±SD, median, and range for continuous variables, and frequency and percentage for categorical variables.

Patient demographics were compared between “rapid” progressors and “slow” progressors, using the two-sample, rank-sum test for continuous variables, Fisher's exact test and a chi^2^ test for categorical variables. The time elapsed between the beginning of symptoms and the first consult either at our Institute or another Hospital was recorded. For survival analysis, “time zero” was defined in two ways: (1) from the beginning of symptoms as reported by the patient; (2) from the “index visit”, which was defined as the date the patient was first seen at the National Institute of Respiratory Diseases during the study period (1995 to 2004). Cumulative survival probabilities were estimated using the Kaplan-Meier method. In addition, a Cox proportional hazards regression model was used to compare survival as a function of several variables including age, gender, clubbing, forced vital capacity (FVC) as percentage of predicted, PaO2, SpO2 at rest and after exercise, smoking (current and cumulative as pack-years) and duration of symptoms before diagnosis. In all cases, two-tailed p values <0.05 were considered statistically significant.

## Results

In univariate analysis of the whole cohort (n = 167), time elapsed between the beginning of symptoms, smoking, masculine gender, FVC%, PaO2, SpO2 at rest and during exercise, were significant predictors of mortality. In the multivariate Cox model, time elapsed between the beginning of symptoms and the first consult, smoking, masculine gender, and FVC%, remained significant.

**Figure 1:**
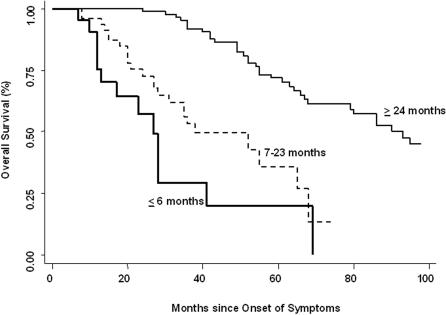
Survival rate. Kaplan-Meier plot of cumulative survival of the whole cohort (n = 167) divided into three groups by the time of the onset of symptoms (≤6 months; 7 to 23 months; and ≥24 months).

Analysis of survival of the whole cohort (n = 167) showed that patients consulting shortly after the beginning of symptoms (≤6 months, n = 26) displayed a worse survival compared with those with ≥24 months of symptoms (n = 88) or intermediate (7–23 months, n = 53) (p<0.001 and p = 0.045 respectively; **figure 1**). Demographic and clinical data of the three groups are shown in **table 1**. The remainder of this analysis is focused on two subpopulations: patients with ≤6 months of symptoms (“rapid” progressors) and patients with ≥24 months of symptoms (“slow” progressors).

**Table 1:** Baseline demographic, clinical, physiologic and BAL characteristics of the study groups as initially diagnosed

Variable	Rapid progressors (n = 26)	Intermediate group (n = 53)	Slow progressors (n = 88)	P**
Age	64.1±12.1	64.3±8.5	63.6±8.8	NS
Gender (male/female)	24/2	38/15	57/31	OR: 6.5 (CI: 1.4–29.4; p = 0.007)
Duration of symptoms before diagnosis (months)	3.6±1.4	13.1±3.2	45.9±22.0	<0.0001
Clubbing (Yes/No)	18/8	41/12	73/15	NS
Smoking status	20/26	27/53	46/88	OR: 3.04 (CI 1.1–8.3; p = 0.04)
Former	15	23	44	
Current	5	4	2	OR: 7.1 (CI: 1.2–40.9; p = 0.01)
Smoking (pack/year)	15.8±16	19.4±24.8	21.1±33.2	NS
**Lung Function Testing^¥^**				
FVC% predicted	58.4±21.6	62±19	61.5±18.1	NS
PaO_2 _mmHg*	48.8±9.1	50±9.1	48.0±10.3	NS
SpO2 rest	85.6±7.6	84.9±12.1	82.0±10.4	NS
SpO2 exercise	73.7±8.0	75.3±9.4	71.0±8.0	NS
**Bronchoalveolar Lavage**				
Total Cell Count (10^5^/ml)	2.7±1.6	2.6±1.9	2.4±1.3	NS
BAL Macrophages (%)	78.4 ±13.3	83.5±8.7	79.7±10.7	NS
BAL Lymphocytes (%)	14.6±7.9	11.7±6.9	14.3±8.5	NS
BAL Neutrophils (%)	3.5±6.2	3.1±3.7	3.8±4.3	NS
BAL Eosinophils%	3.2±2.8	1.7±1.4	2.3±2.8	NS
HRCT extent of ground glass attenuation	0.8±0.4	0.9±0.7	0.7±0.5	NS
HRCT extent of reticular lesions^§^	1.9±0.6	1.8±0.4	1.8±0.6	NS

FVC: forced vital capacity; SpO_2_: oxygen saturation; BAL: bronchoalveolar lavage; HRCT: high resolution computed tomography.

* Normal values at Mexico City altitude: 67±3 mmHg.

** “Rapid” and “slow” progressors were compared with a T test for independent groups in case of continuous variables or with a chi square test or Fisheŕs exact test for count variables.

For the “rapid” progressors, the median follow-up time from beginning of symptoms was 13.5 months (CI: 11.5–23.5) and the median survival was 27 months (CI: 13–41). For the “slow” progressors, the median follow-up time from beginning of symptoms was 60.5 months (CI: 52–69.4), and the median survival was 93 months (CI: 68–105). When examined from the time of diagnosis, the “rapid” progressors had a median follow-up time of 10 months (CI: 7.4–21), while the median survival was 25 months (CI: 10–38). For the “slow progressors”, the median follow-up time from diagnosis was 17 months (CI: 13–22) and the median survival was 32 months (CI: 25–42).

No differences in age, lung functional alterations, oxygen saturation, extent of HRCT changes and BAL cell profile were found between the groups (**Table 1**). Likewise, no differences were observed in the presence/absence of emphysematous lesions on HRCT: 19% in the rapid progressors and 27.4% in the “slow” progressors, p = 0.57. Also, there were no differences in the socioeconomic and educational background, which may influence the promptness to consult by symptoms (data not shown). There was a significant increase of males (OR = 6.5; CI: 1.4–29.5; p = 0.006), ever smokers (OR = 3.04; CI: 1.1–8.3; p = 0.04) and current smokers (OR = 7.1, CI: 1.2–40.9; p = 0.02) in the group of “rapid” progressors. No difference was found in the smoking index (**Table 1**).

### Histopathology

Thirty one percent of the patients were biopsied (8/26 “rapid” and 27/88 “slow” progressors). None of the patients had been treated with corticosteroids or immunosuppressive drugs at the time of biopsy. Among those biopsied, no differences were found in the morphologic parameters explored: interstitial inflammation, pulmonary hypertension changes, smooth muscle hyperplasia, type 2 cell hyperplasia, and extent of fibrosis or honeycombing. Hyaline membranes were not observed. “Rapid” progressor patients showed 6.9±4.1 FF/cm^2^ versus 4.9±3.8 FF/cm^2^ from “slow” progressors (p = 0.2).

### Survival rate

We obtained the vital status on 85% of the “rapid” progressors and of 80% of the “slow” progressors. For the remaining patients, their vital status was considered alive. As illustrated in **Figure 1**, survival rate determined from the beginning of symptoms was significantly lower in the “rapid” progressors group (Hazard ratio = 9.0; CI: 4.48–18.3; p<0.0001). Mortality determined from the time of diagnosis (at the “index visit”) showed a tendency to be increased in the “rapid” progressors group although it did not reach statistical significance (HR = 1.5; CI 0.81–2.87; p = 0.18).

### Six months follow-up

**Figure 2:**
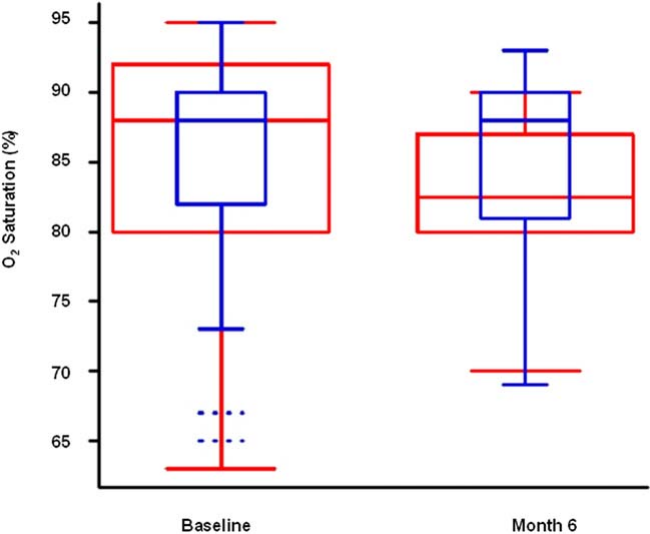
Distributions of oxygen saturation at rest at baseline and after 6 months follow-up. Box-and-whisker plots show the distributions for “rapid” progressors (red) and “slow” progressors (blue). Boxes indicating the middle 50% of data points extend from the first (25%) quartile to the third (75%) quartile. The height of each box is the interquartile range (IQR). The second (50%) quartile, or median, is indicated by a line within each box. Whiskers extend out to the smallest and largest data points within 1.5 IQRs of the first and third quartiles, respectively. Observations beyond the whiskers are potential outliers, indicated here by only two dashed lines, among slow progressors at baseline.

At 6 months follow-up, no differences were found in FVC [Rapid progressors: 60.9±17.2%; slow progressors: 62.7±17.9%]. However, rapid progressors showed a significant reduction of SpO_2_ at rest. As shown in figure 2, there was no difference in the distribution of SpO_2_ between rapid and slow progressors at baseline (p = 0.90) but at 6 months follow-up, 75% of rapid progressors had oxygen saturation values below the median of slow progressors. ANCOVA model with 6-month oxygen saturation as response, and adjusting for baseline oxygen saturation revealed a significant difference between rapid and slow progressors (p = 0.027).

### Transcriptional profiling suggests a difference between “rapid” and “slow” progressors

Gene expression profiles were obtained in 8 patients who fulfilled the criteria for “rapid” (n = 4) or “slow” (n = 4) progressors. Rapid progressors (59±6.5 years) were all male, one of them current and two former smokers. Slow progressors (61±7.0) were also male, and two of them were former smokers. These patients showed no differences in pulmonary function tests and BAL cell profile (data not shown). These subjects were included in a previous analysis of gene expression; however, the data presented here were not reported in the earlier study [13].

**Figure 3:**
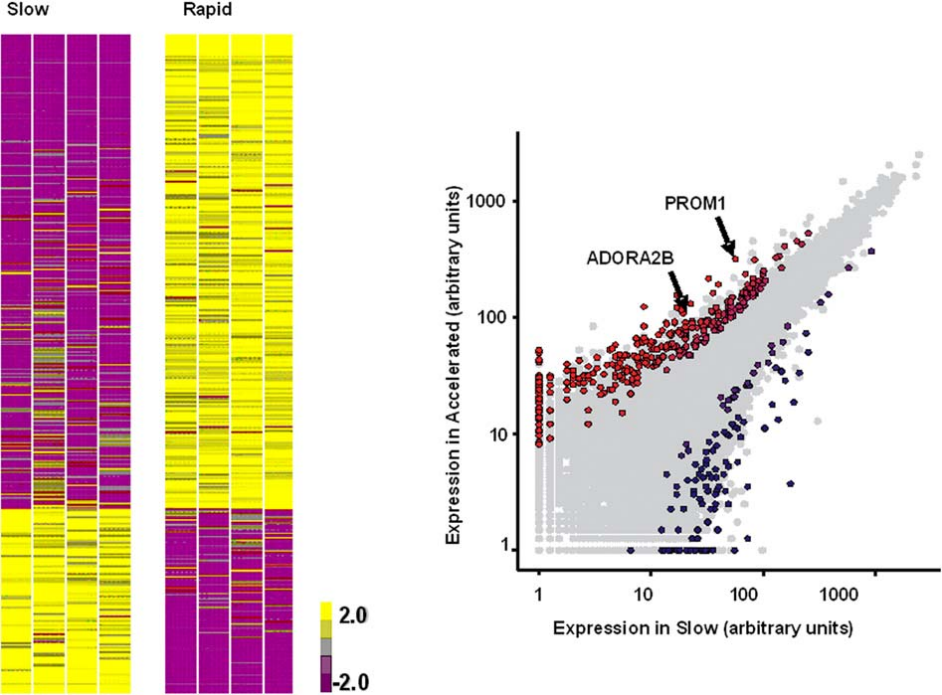
Gene expression patterns that distinguish “rapid” progressors from “slow” progressors. (A) Infogram of 437 differentially expressed genes in individual rapid and slow progressor patients. The expression levels for each gene were normalized to the geometric mean of all the samples for each gene. Increased genes are shown in progressively brighter shades of yellow, and decreased genes are shown in progressively darker shades of blue. Genes shown in gray are not different between the groups. The genes were ranked according to their significance level. (B) A log scale scatter plot of the average of intensity of all the genes on the arrays in rapid progressors (X-axis) and slow progressors (Y-axis). Colored points-437 genes that were significantly changed (p-value <0.05 in TNoM and t-test and fold ratio >2). Points are colored by their fold ratios; progressive shades of blue indicate increase and progressive shades of red indicate decrease. Points colored in gray did not reach significance. Adenosine A_2B_ receptor (ADORA2B) and prominin-1/CD133 (PROM1) were among the most upregulated genes in the “rapid” progressor group.

We could not find genes with a false discovery rate [21] of less than 20% for TNoM, t-test or SAM [22]. Therefore we have defined genes as substantially differentially expressed if they had a t-test and a TNoM p-value <0.05 and a fold ratio >2 as previously described [20]. There were 1036 genes with a p-value <0.05 for TNoM and 1645 for t-test. The intersection of both reduced the gene number to 801 and the requirement for a 2 fold change reduced the number to 437 genes that were substantially upregulated or downregulated in “rapid” progressors compared to “slow” progressors (Figure 3). “Rapid” progressor patients strongly expressed genes involved in morphogenesis, cancer, oxidative stress, apoptosis, cell migration/proliferation, and genes from fibroblasts/smooth muscle cells (Table S1). Around 30% of the differentially expressed genes were downregulated in the rapid progressor lungs, including genes related to signal transducer activity, and epithelial receptors among others (Table S2).

### Immunolocalization of adenosine-2B receptor (A_2B_AR) and prominin-1/CD133

**Figure 4:**
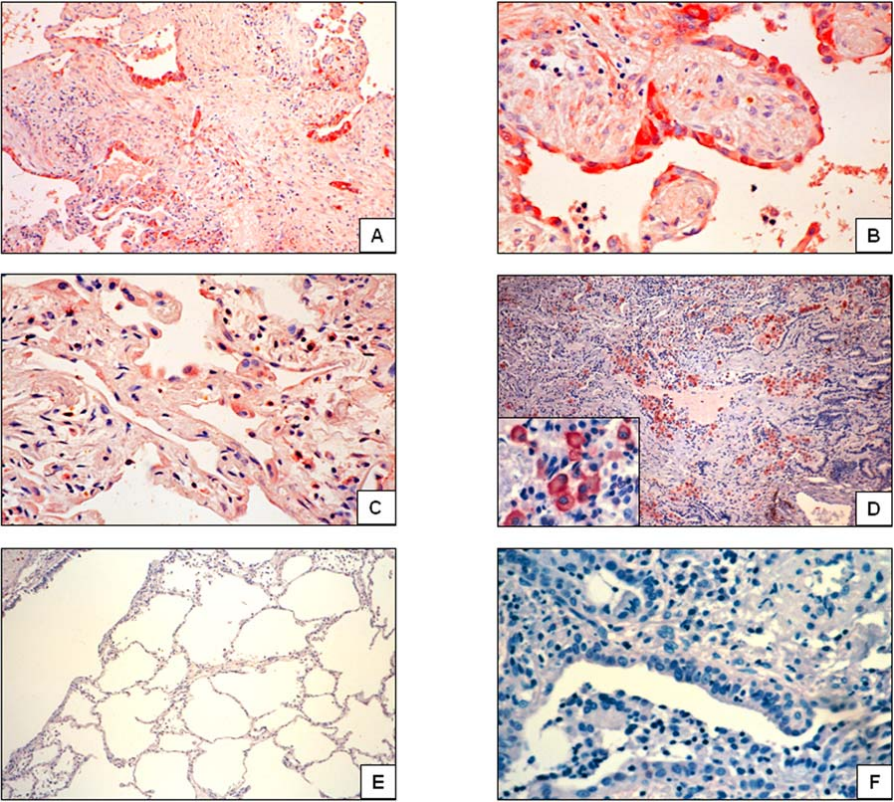
Localization of adenosine A_2B_ receptor in IPF lungs. Immunoreactive protein was revealed with 3-amino-9-ethyl-carbazole and samples were counterstained with hematoxylin. Panels A and B show two different IPF lungs from rapid progressors exhibiting strong epithelial staining of A_2B_ AR (original magnification, 10 and 40×). Stained fibroblasts are also seen in panel B. Panel C: A_2B_ AR staining in an IPF lung from slow progressor. Panel D: Lung specimen from hypersensitivity pneumonitis displaying positive macrophage staining for A_2B_ AR (10×, inset 40×). Panel E: Control lung (10×). Panel F: Negative control section from IPF lung in which the primary antibody was replaced with non-immune serum (40×).

The cellular source of A_2B_AR and prominin-1, two highly upregulated genes in “rapid” progressor patients, was determined by immunohistochemistry in “rapid” and “slow” progressor IPF lungs as well as in hypersensitivity pneumonitis and normal control lungs. In IPF lungs from “rapid” progressors, the immunoreactive A_2B_AR protein was strongly expressed in reactive alveolar epithelial cells and fibroblasts (**Figure 4A and 4B**). Less intense staining was observed in “slow” progressors (**Figure 4C**). Lungs from patients with hypersensitivity pneumonitis showed staining primarily in alveolar macrophages (**Figure 4D**). Control lungs showed scattered positive cells for A_2B_AR.

**Figure 5:**
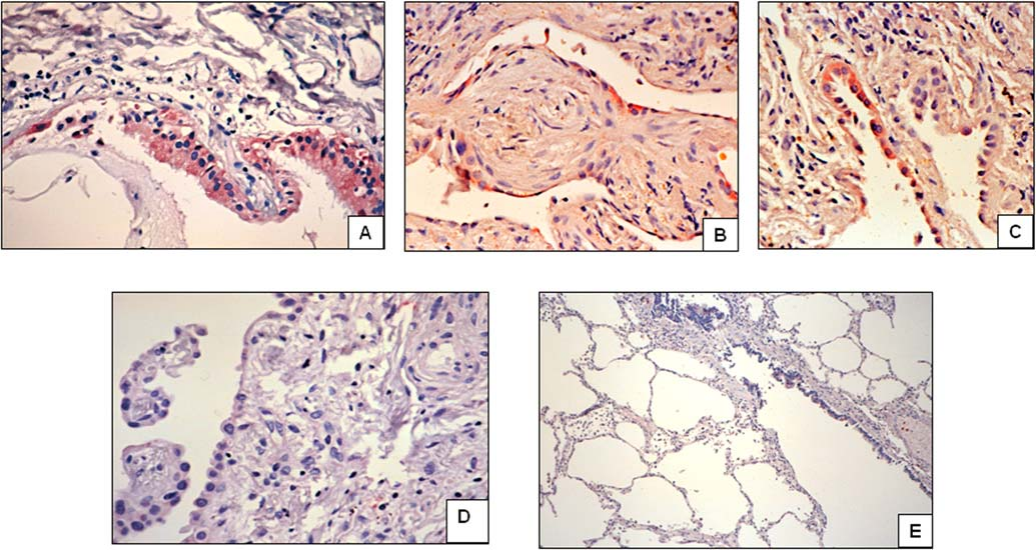
Immunolocalization of prominin-1/CD133 in IPF lungs. Panels A, B, and C: Three different IPF lungs from “rapid” progressor patients showing staining in an area of bronchiolar metaplasia (A) and in reactive alveolar epithelial cells (B and C). Panels D and E: “Slow” progressor IPF lung (D) and normal control (E) showing no staining.

Prominin-1/CD133 was detected mostly in the lungs of “rapid” progressor patients (5 from 7). The immunoreactive protein was observed in some areas of hyperplastic type 2 pneumocytes and attenuated alveolar epithelial cells covering fibroblastic foci as well in some areas of bronchiolar metaplasia (**Figure 5A–C**). By contrast, with the exception of one patient that showed occasional positive cells, in “slow” progressor lungs prominin was virtually absent (**Figure 5D**). Lungs from patients with hypersensitivity pneumonitis and normal lungs were negative for prominin-1/CD133 signal as exemplified in a control lung in **Figure 5E**.

### Immunoblotting

**Figure 6:**
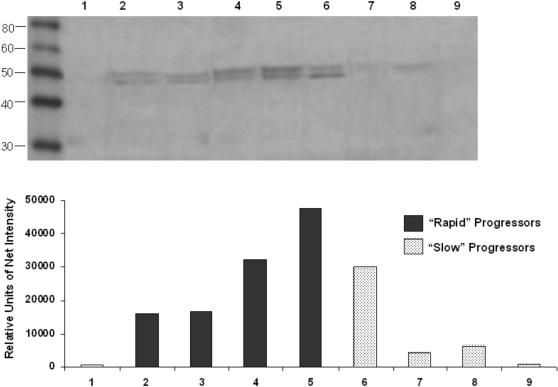
Immunoblotting of adenosine-2B receptor. Top: Western blot analysis of BAL fluid proteins (35 µg/line) using an anti-A_2B_ receptor antibody. Samples were from normal individual (lane 1), “rapid” progressors (lanes 2–5) and “slow” progressors (lanes 6–9). Bottom: Quantitative densitometry of the bands shown in top panel.

Western blot analysis and quantitative densitometry of the adenosine-2B receptor in BAL fluids are shown in **figure 6**. Samples from healthy individuals were usually negative (lane 1). By contrast, a double band of ∼50 kDa was observed in most of the IPF samples. BAL samples from “rapid” progressors showed stronger immunoreactivity compared with slow progressors. Prominin was not detected in BAL fluids.

### Fibroblast migration

**Figure 7:**
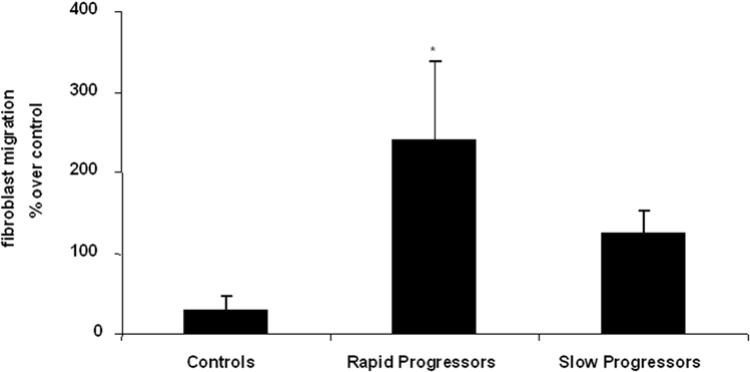
Fibroblast migration. Fibroblasts were placed in the upper compartment of a Boyden-type chamber, and F-12 medium containing 5% BSA alone or with 50% BAL fluid was added to the lower compartment. After 8 h of incubation, the migrating cells were stained and the absorbance of the stained solution was measured by ELISA. *p<0.01 versus controls and p<0.05 versus slow progressors.

Several genes related to cell migration were upregulated in the “rapid” progressors' lungs (**Table S1**). Therefore, we determined if BAL fluids from these patients affected fibroblast migration. For this purpose, we evaluated 6 “slow” progressors and 6 “rapid” progressors BAL samples from the same cohort of patients, as well as 5 normal individuals. Selected patients from both groups of patients were similar in age and pulmonary function abnormalities, and included former and nonsmoker cases. Human lung fibroblasts were exposed to BAL fluids and cell migration was evaluated in collagen-coated Boyden chambers. The number of fibroblasts that migrated in absence of BAL was used as control (0% migration). As illustrated in **figure 7**, BAL from “rapid” progressors induced a significant increase in fibroblast migration compared with BAL from “slow” progressors and from healthy controls [238±98% versus 123±29% (p<0.05) and 30±17% (p<0.01) respectively].

### Gelatin zymography of BAL fluids

**Figure 8:**
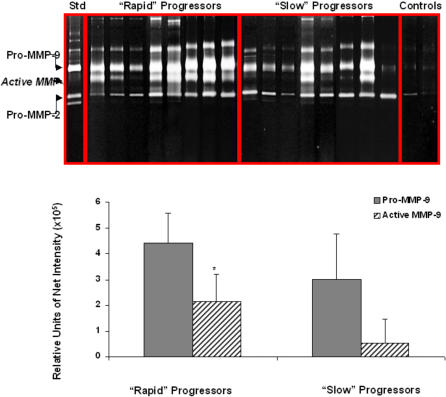
Identification of gelatinolytic activities in bronchoalveolar lavage from controls, and rapid and slow progressors IPF patients. Supernatants were resolved by SDS-PAGE gels (8.5%) containing gelatin (1 mg/ml) and a final concentration of 0.3 mg/ml heparin. Std = MMP-9 and MMP-2 zymography standards. The relative variations of gelatinase levels were analyzed by densitometry. Densitometric analysis of pro-MMP-9 and active MMP-9 is shown in the bottom. *P<0.05.

To identify possible differences in BAL gelatinolytic activities, aliquots containing 1.5 µg of protein from 8 “rapid” progressors, 8 “slow” progressors and 7 controls were analyzed by gelatin substrate gel zymography. Selected patients from both groups were similar in age and pulmonary function abnormalities, and included former and nonsmoker cases. A representative zymogram comparing “rapid” progressors with “slow” progressors is shown in **figure 8**. BAL control samples showed faint bands of 72 kDa and 92 kDa activities corresponding to progelatinase A and progelatinase B respectively. BAL fluids obtained from rapid progressors showed a significant increase of the activated 86 kDa form of the MMP-9. Densitometric analysis corroborated a >2-fold increase of gelatinase B activity in comparison with slow progressors (p<0.05). No differences were found in progelatinases and gelatinase A activities. All gelatinolytic bands were fully inhibited by 10 mM EDTA (not shown).

## Discussion

In this study, we evaluated the clinical behavior and survival rate of IPF patients classified as “rapid” or “slow” progressors according to the duration of symptoms before diagnosis. Our results showed that patients consulting within 6 months after the beginning of symptoms were primarily males who smoked. Moreover, gas exchange deteriorates faster in “rapid” progressors and their survival was appreciably worst compared to those patients who presented with more than 2 years duration of symptoms prior to diagnosis. Using microarrays, tissue protein verification and BAL analysis, we show that “rapid” progressors appear to represent a distinct biological phenotype among patients with IPF. Indeed, “rapid” progressors were not patients presenting “earlier” or with milder disease but were similar in the degree of physiologic, radiologic and histopathologic abnormalities as those subjects with longer periods of clinical symptoms.

### Evidence of a Subset of IPF with Fulminant Disease

There are a number of diseases in which major differences in the phenotypic behavior have been described, e.g., rate of progression of HIV infection [23]; severity and rate of progression to liver fibrosis after liver transplantation for end-stage disease caused by chronic hepatitis C virus infection [24]; or progression to end-stage renal failure in patients with focal segmental glomerulosclerosis [25]. Also, the rate of decline of lung function in healthy smokers who continue to smoke is heterogeneous, and a subgroup of them exhibits an accelerate rate of decrease of FEV_1_ while others progress slowly or even show no decline at all [26]. In all of these examples, the reasons for the variability in the clinical expression of the disease are unknown but a number of modifying genes or environmental factors might be responsible.

Clinical observations have suggested that IPF patients exhibit variable clinical courses. In addition, it has recently been emphasized that patients with established IPF may suffer “acute exacerbation” of their disease defined as a clinical syndrome characterized by sudden worsening of dyspnea, newly developing diffuse radiographic opacities, worsening hypoxemia, and absence of infectious pneumonia, heart failure, or sepsis [5], [6]. This process is characterized by diffuse alveolar damage on the background of the otherwise typical UIP pattern of injury found in IPF. The rapidly progressive form of IPF that we describe in this study does not correspond to an acute exacerbation of IPF. We propose that the “rapid” progressors described here represent a variant of chronic IPF. Gender and smoking status were associated with this variant although the putative mechanism by which they may be implicated in the “rapid” progression phenotype is unknown.

Pulmonary function tests and HRCT scanning have been proposed to estimate disease severity and monitor disease progression [27], [28]. It is tempting to consider the time of symptoms before the first consult (if extreme, i.e., less of 6 months) as indication of early disease. However, our findings indicate that the duration of symptoms before diagnosis is not a useful parameter to classify the stage of IPF as early or advanced. The physiologic and radiographic features were not different between the “rapid” and “slow” progressors. Further, our results suggest that patients probably consult when the severity of the lung lesions reaches a threshold that is enough to provoke symptoms, and this can occur rapidly or slowly.

### Rapid Progressors Seem to Differ Biologically from Slow Progressors

Global gene expression analysis performed in a subset of patients identified a number of genes that were differentially expressed in both groups. Although the analyzed sample is small, and the statistical power is limited, the upregulation of several functional pathways became apparent in the lungs from rapid progressor patients. These pathways seem to reflect diverse molecular mechanisms mostly operating in alveolar epithelial and mesenchymal cells and include genes involved in cell motility, myofibroblast differentiation, oxidative stress, coagulation and development. Although we can not ruled-out some effect of smoking, the differentially expressed genes among “rapid” and “slow” progressors found in this work differ from those described as associated to smoking [29]–[32].

Of the genes increased in rapid progressors we chose to verify and localize two of them. One was the adenosine A_2B_ receptor gene, which is involved in the differentiation of human lung fibroblasts to myofibroblasts-a key process in fibrotic remodeling [33]. Interestingly, partially adenosine deaminase-deficient mice show upregulation of this receptor and exhibit spontaneous and progressive pulmonary fibrosis and usually die from respiratory distress [34]. The immunoreactive A_2B_AR was mainly observed in alveolar epithelial cells and fibroblasts in “rapid” progressor lungs. Taken together, these results suggest a potential regulatory role for adenosine or its receptor in IPF. Another intriguing gene increased in the “rapid” progressor lungs was prominin-1/CD133, which is found in hematopoietic stem cells and embryonic epithelium among others [35], [36]. The immunoreactive protein was also expressed by reactive alveolar epithelial cells of “rapid” progressor lungs, supporting the notion that these cells may undergo a phenotypic shift during the pathogenesis of IPF. Genes related to the coagulation pathway were increased in “rapid” progressors. Three of them, beta3-endonexin, serine protease inhibitor, Kazal type, and plasminogen activator inhibitor-1 are antifibrinolytic and may enhance fibrin deposition, a process that has been associated with fibrosis [3]. Interestingly, two genes associated with the Hermansky-Pudlak syndrome, HPS3 and Rab38, a small GTPase of the Rab family expressed in bronchial and alveolar epithelial cells, were increased in the “rapid” progressors group. Hermansky-Pudlak syndrome is a genetic disorder characterized by oculocutaneous albinism, a bleeding diathesis, and in a subset of patients, pulmonary fibrosis [37].

Decreased genes in “rapid” progressors included Smad6, an inhibitor of TGF-beta Smad-mediated signal transduction, and the receptor for advanced glycation end-products which is a highly selective differentiation marker of alveolar epithelial type I cells [38]. This receptor is downregulated in bleomycin induced lung fibrosis [39]. Also decreased in “rapid” progressors were a disintegrin-like and metalloprotease with thrombospondin type 1 motif, 7 (ADAMTS7), the chemokine receptor CXCR6, and Bcl2-L-10, a novel anti-apoptotic member of the Bcl-2 family. The downregulation of Smad6, an inhibitor of TGF-β signaling, together with the increase in plasminogen activator inhibitor-1 (a universal TGF-β responsive gene) may signify disregulated TGF-β-mediated fibrotic response.

“Rapid” progressors also showed higher levels of active MMP-9 in the BAL fluids. Excessive MMP-9 activity may contribute to the loss of integrity of the basement membranes and subsequently to abnormal tissue repair [13], [40]–[42]. Cell surface-localized MMP-9 is also able to activate TGF-β providing a potential mechanism for the aberrant tissue remodeling [43]. As mentioned, this effect may be potentiated by the downregulation of Smad 6 revealed in “rapid” progressors IPF patients. Activation of MMP-9 can be achieved by oxidative stress, and interestingly, several genes associated with this process were upregulated in “rapid” progressors. For example, CYP2F1, which encodes a cytochrome P450 enzyme capable of bioactivating a number of pulmonary-selective toxicants producing highly reactive metabolites, was significantly increased [44].

The limited size of our microarray sample does not support the use of the genes identified as biomarkers that distinguish rapid and slow patients. However, the relatively stringent selection of genes, the protein verification by immunohistochemistry on additional samples and the biological relevance of the genes suggest that our results are biologically meaningful. Taken together with the analysis of the biological activity of BAL and evidence for MMP-9 activation our results provide evidence that the two clinical phenotypes that we identified are also biologically distinct. Additionally, our findings suggest that molecular changes can be more sensitive than morphology or radiology to find moderate/subtle changes.

In conclusion, our study represents the first identification of a distinct clinical phenotype of IPF–one that differs in clinical course and transcriptional profile, despite having similar lung function, chest imaging, and histology findings. Our findings suggest that during the development of this complex disease, genetic modifiers (and environmental factors, i.e. smoking) may play an important role in determining the eventual clinical phenotype, and that some modifiers genes are inducing a more aggressive IPF phenotype. We are aware that our study has limitations: the retrospective data collection, the dependence on patient reporting of the duration of symptoms, and the small number of tissues available for microarray analysis. However, the relatively large number of studied patients, our ability to confirm the results of gene expression by immunohistochemistry and the convincing BAL data, support the validity of these observations. Taken together with reports of the impact of acute exacerbations of IPF on morbidity and mortality, our results further highlight the variability in the progression and outcome of IPF. These findings may explain, in part, the difficulty in obtaining significant and reproducible results in studies of therapeutic interventions in patients with IPF [45] and support prospective studies to identify highly reproducible biomarkers for disease progression and outcome.

## Supporting Information

Table S1 Upregulated Genes in Rapid Progressors
(0.44 MB DOC)

Table S2 Downregulated Genes in Rapid Progressors
(0.16 MB DOC)
