# Supplementary material for: Accelerated Variant of Idiopathic Pulmonary Fibrosis: Clinical Behavior and Gene Expression Pattern
Source: PLoS One. 2007 May 30;2(5):e482. doi: 10.1371/journal.pone.0000482 (PMC1868965; doi:10.1371/journal.pone.0000482)
Supplement: Table S1 — Upregulated Genes in Rapid Progressors (0.44 MB DOC) [file pone.0000482.s001.doc]

## Table S1

## Upregulated Genes in Rapid Progressors

| **GenBank Accn Locus Link** | **Gene** | **TNOM PValue** | **t-Test PValue** | **Fold Change**  **(log base 2)** |
| --- | --- | --- | --- | --- |
| 10641 | Tumor suppressor candidate 4 (TUSC4) | 0.0285714 | 3.40E-06 | 5.61006 |
| 157657 | Hypothetical protein (LOC157657) | 0.0285714 | 1.19E-05 | 5.2329 |
| 10611 | LIM protein (similar to rat protein kinase C-binding enigma) | 0.0285714 | 4.85E-05 | 5.19984 |
| 119392 | Chromosome 10 open reading frame 78 | 0.0285714 | 5.28E-06 | 4.88344 |
| 5270 | Serine (or cysteine) proteinase inhibitor, clade E (SERPINE2) | 0.0285714 | 0.00411436 | 4.78758 |
| 83661 | Membrane-spanning 4-domains, subfamily A, member 8B | 0.0285714 | 0.0168858 | 4.77141 |
| 124045 | Hypothetical protein (FLJ31606) | 0.0285714 | 6.23E-06 | 4.70858 |
| 5553 | Proteoglycan 2, bone marrow (natural killer cell activator, eosinophil granule major basic protein) | 0.0285714 | 6.13E-07 | 4.70468 |
| 25924 | Myosin VIIA and Rab interacting protein (MYRIP) | 0.0285714 | 0.010653 | 4.68344 |
| 1573 | Cytochrome P450, family 2, subfamily J, polypeptide 2 (CYP2J2) | 0.0285714 | 0.009126 | 4.6235 |
| 285237 | Hypothetical protein (MGC26717). | 0.0285714 | 4.64E-07 | 4.58434 |
| 55246 | Hypothetical protein (FLJ10853). | 0.0285714 | 4.87E-05 | 4.57228 |
| 29968 | Phosphoserine aminotransferase 1 (PSAT1), transcript variant 1 | 0.0285714 | 5.33E-05 | 4.54582 |
| 57185 | Hypothetical protein dJ462O23.2 (DJ462O23.2) | 0.0285714 | 0.0002491 | 4.53408 |
| 7014 | Telomeric repeat binding factor 2 | 0.0285714 | 0.000305488 | 4.52766 |
| 79594 | Hypothetical protein (FLJ12875) | 0.0285714 | 6.48E-06 | 4.52284 |
| 8678 | Hypothetical protein (FLJ40137), | 0.0285714 | 0.000368106 | 4.51186 |
| 64840 | Likely ortholog of mouse porcupine homolog (Drosophila) (PPN) | 0.0285714 | 0.00653064 | 4.50807 |
| 5304 | Prolactin-induced protein | 0.0285714 | 0.0462135 | 4.50718 |
| 2296 | Forkhead box C1 | 0.0285714 | 0.00026266 | 4.48807 |
| 22847 | Zinc finger protein 507 | 0.0285714 | 0.00468148 | 4.48393 |
| 23059 | Clusterin associated protein 1 | 0.0285714 | 0.00648649 | 4.47824 |
| 94025 | Mucin 16 | 0.0285714 | 0.00354 | 4.4642 |
| 6730 | Signal recognition particle 68kDa (SRP68) | 0.0285714 | 3.73E-07 | 4.45784 |
| 9319 | Thyroid hormone receptor interactor 13 | 0.0285714 | 0.00862173 | 4.4568 |
| 55259 | Cancer susceptibility candidate 1 (CASC1) | 0.0285714 | 0.00614876 | 4.43344 |
| 112752 | MGC16028 similar to RIKEN cDNA 1700019E19 gene | 0.0285714 | 0.00225395 | 4.40717 |
| 2729 | Glutamate-cysteine ligase, catalytic subunit | 0.0285714 | 0.00074083 | 4.3997 |
| 205 | Adenylate kinase 3 | 0.0285714 | 0.0241213 | 4.3891 |
| 56986 | Hs.127432:x 009 protein | 0.0285714 | 0.000277768 | 4.36916 |
| 5074 | PRKC, apoptosis, WT1, regulator. | 0.0285714 | 0.00117179 | 4.33808 |
| 84071 | Hypothetical protein (DKFZP434P0714) | 0.0285714 | 0.000689762 | 4.31758 |
| 79885 | Histone deacetylase 11 | 0.0285714 | 0.00118705 | 4.26607 |
| 10158 | Membrane-associated protein 17 (MAP17) | 0.0285714 | 0.000208528 | 4.25701 |
| 78999 | Hypothetical protein (MGC3103) | 0.0285714 | 0.000346922 | 4.2544 |
| 84951 | C-terminal tensin-like (CTEN) | 0.0285714 | 0.0370227 | 4.24708 |
| 23408 | Sirtuin (silent mating type information regulation 2 homolog) 5 (S. cerevisiae) (SIRT5) | 0.0285714 | 1.81E-05 | 4.24122 |
| 57805 | P30 DBC protein (DBC-1), transcript variant 1 | 0.0285714 | 0.000758958 | 4.21802 |
| 339327 | Hypothetical protein (MGC43537) | 0.0285714 | 0.00160202 | 4.21232 |
| 284804 | Similar to dJ1184F4.4 (novel protein similar to nucleolar protein 4 (NOL4) | 0.0285714 | 0.00095868 | 4.20344 |
| 4289 | Muskelin 1, intracellular mediator containing kelch motifs | 0.0285714 | 0.00729531 | 4.20257 |
| 23421 | Integrin beta 3 binding protein (beta3-endonexin) | 0.0285714 | 0.00159417 | 4.18731 |
| 11157 | LSM6 homolog, U6 small nuclear RNA associated (S. cerevisiae) | 0.0285714 | 0.000165458 | 4.17366 |
| 79978 | CD22 antigen (CD22) | 0.0285714 | 0.0112136 | 4.17259 |
| 57711 | KIAA1615 protein | 0.0285714 | 0.000759004 | 4.15162 |
| 140460 | Ankyrin repeat and SOCS box-containing 7 (ASB7). | 0.0285714 | 0.00570916 | 4.12591 |
| 56995 | Tubby like protein 4 (TULP4) | 0.0285714 | 0.00393737 | 4.12498 |
| 286826 | TUDOR gene similar (TGS) | 0.0285714 | 8.40E-05 | 4.12472 |
| 136 | Adenosine A2b receptor (ADORA2B) | 0.0285714 | 0.0350408 | 4.12032 |
| 55350 | Vanin 3 (VNN3), transcript variant 1 | 0.0285714 | 0.0097086 | 4.11083 |
| 5333 | Phospholipase C, delta 1 (PLCD1) | 0.0285714 | 0.000075857 | 4.10238 |
| 220929 | Hypothetical protein (LOC220929) | 0.0285714 | 0.00200293 | 4.09879 |
| 64123 | EGF, latrophilin and seven transmembrane domain | 0.0285714 | 0.000936619 | 4.0865 |
| 9508 | A disintegrin-like and metalloprotease (reprolysin type) with thrombospondin type 1 motif, 3 (ADAMTS3) | 0.0285714 | 0.000926503 | 4.06568 |
| 8575 | Protein kinase, interferon-inducible double stranded RNA dependent activator (PRKRA) | 0.0285714 | 0.00134858 | 4.05491 |
| 10466 | Component of oligomeric golgi complex 5, transcript variant 1 | 0.0285714 | 0.000437857 | 4.04528 |
| 5510 | Protein phosphatase 1, regulatory subunit 7 | 0.0285714 | 0.000311845 | 4.03952 |
| 8842 | Prominin 1 | 0.0285714 | 0.0410778 | 4.03758 |
| 8833 | Guanine monophosphate synthetase | 0.0285714 | 0.0119575 | 4.03601 |
| 27300 | Zinc finger protein (AF020591) | 0.0285714 | 0.000484719 | 4.00776 |
| 130367 | Sphingosine-1-phosphate phosphatase 2 | 0.0285714 | 0.0112611 | 3.99951 |
| 84343 | Hermansky-Pudlak syndrome 3 | 0.0285714 | 0.0294907 | 3.99595 |
| 63921 | Helicase, ATP binding 1 (HELIC1) | 0.0285714 | 0.00893226 | 3.99166 |
| 10606 | Phosphoribosylaminoimidazole carboxylase | 0.0285714 | 0.000125132 | 3.99054 |
| 84163 | Transcription factor GTF2IRD2 (GTF2IRD2). | 0.0285714 | 0.0118648 | 3.98532 |
| 55135 | Hypothetical protein (FLJ10385) | 0.0285714 | 0.00196059 | 3.9828 |
| 8548 | Basic leucine zipper nuclear factor 1 (JEM-1) (BLZF1) | 0.0285714 | 0.0135431 | 3.9646 |
| 137492 | Hepatocellular carcinoma related protein 1 | 0.0285714 | 0.00114464 | 3.95197 |
| 84749 | Ubiquitin specific protease 30 | 0.0285714 | 0.00724906 | 3.9352 |
| 4113 | Melanoma antigen, family B, 2 (MAGEB2). | 0.0285714 | 0.00248247 | 3.93268 |
| 79646 | Pantothenate kinase 3 | 0.0285714 | 0.000764675 | 3.92162 |
| 26973 | Cysteine and histidine-rich domain (CHORD)-containing, zinc binding protein 1 | 0.0285714 | 0.0171402 | 3.91936 |
| 55709 | Kelch repeat and BTB (POZ) domain containing 4, transcript variant 2 | 0.0285714 | 0.000797307 | 3.89296 |
| 9182 | Peptidylglycine alpha-amidating monooxygenase COOH-terminal interactor | 0.0285714 | 0.0163587 | 3.89068 |
| 51175 | Tubulin, epsilon 1 (TUBE1) | 0.0285714 | 0.00437776 | 3.88672 |
| 79657 | Hypothetical protein FLJ21908 | 0.0285714 | 0.0114617 | 3.86383 |
| 138162 | Hypothetical protein (MGC29761) | 0.0285714 | 0.0456348 | 3.85153 |
| 253769 | Hypothetical protein (MGC43690) | 0.0285714 | 0.00826743 | 3.84507 |
| 203523 | Hypothetical protein (FLJ23614) | 0.0285714 | 0.00289595 | 3.84484 |
| 55779 | Hypothetical protein (FLJ11142) | 0.0285714 | 0.020745 | 3.8447 |
| 2720 | Galactosidase, beta 1 (GLB1) | 0.0285714 | 0.00352646 | 3.84362 |
| 2153 | Coagulation factor V (proaccelerin, labile factor) | 0.0285714 | 0.00162304 | 3.84158 |
| 95681 | Testis specific, 14 | 0.0285714 | 0.00392805 | 3.82779 |
| 340533 | KIAA2022 protein | 0.0285714 | 0.0013342 | 3.8248 |
| 9742 | KIAA0590 gene product; intraflagellar transport 140 homolog (Chlamydomonas) | 0.0285714 | 0.000258585 | 3.82352 |
| 56675 | Nuclear receptor interacting protein 3 | 0.0285714 | 0.0022435 | 3.78588 |
| 3313 | Heat shock 70kDa protein 9B (mortalin-2) | 0.0285714 | 0.00577159 | 3.76241 |
| 50999 | Transmembrane emp24 protein transport domain containing 5 | 0.0285714 | 0.00639427 | 3.76198 |
| 23190 | UBX domain containing 2 (UBXD2) | 0.0285714 | 0.000361771 | 3.7368 |
| 253512 | Hypothetical protein LOC253512 | 0.0285714 | 4.88E-06 | 3.72672 |
| 25914 | Rotatin (RTTN) | 0.0285714 | 0.000735404 | 3.72294 |
| 10363 | High-mobility group 20A (HMG20A). | 0.0285714 | 0.0244564 | 3.72186 |
| 51409 | HEMK homolog 7kb (HEMK) | 0.0285714 | 0.0168885 | 3.72137 |
| 79882 | Nuclear protein UKp68 | 0.0285714 | 0.0175414 | 3.71857 |
| 283150 | Hypothetical protein LOC283150 | 0.0285714 | 0.00789392 | 3.71853 |
| 7803 | Protein tyrosine phosphatase type IVA, member 1 | 0.0285714 | 0.0041165 | 3.71836 |
| 284443 | Zinc finger protein 493 (ZNF493). | 0.0285714 | 0.000315841 | 3.70792 |
| 63928 | Hepatocellular carcinoma antigen gene 520 | 0.0285714 | 0.048794 | 3.68864 |
| 11264 | Peroxisomal membrane protein 4, 24kDa (PXMP4), transcript variant 1 | 0.0285714 | 0.000287254 | 3.64328 |
| 133957 | Similar to RIKEN cDNA 0610011N22 (LOC133957) | 0.0285714 | 0.000818486 | 3.63768 |
| 53904 | Myosin IIIA | 0.0285714 | 0.0100176 | 3.61647 |
| 5529 | Protein phosphatase 2, regulatory subunit B (B56), epsilon isoform | 0.0285714 | 0.00179755 | 3.60956 |
| 1572 | Cytochrome P450, family 2, subfamily F, polypeptide 1 (CYP2F1). | 0.0285714 | 0.0179691 | 3.58723 |
| 7298 | Thymidylate synthetase | 0.0285714 | 0.0181964 | 3.57737 |
| 10100 | Tetraspan 2 (TSPAN-2) | 0.0285714 | 0.0182132 | 3.56746 |
| 125488 | Hypothetical protein FLJ33761 | 0.0285714 | 0.000170471 | 3.56464 |
| 11021 | RAB35, member RAS oncogene family | 0.0285714 | 0.0257581 | 3.5602 |
| 54954 | Hypothetical protein (FLJ20506) | 0.0285714 | 0.00762312 | 3.55358 |
| 23057 | Nicotinamide nucleotide adenylyltransferase 2 , transcript variant 1 | 0.0285714 | 0.0311568 | 3.54939 |
| 22954 | Tripartite motif-containing 32 | 0.0285714 | 0.0205441 | 3.54017 |
| 200879 | Lipase, member H (LIPH) | 0.0285714 | 0.00903779 | 3.52838 |
| 168090 | Chromosome 6 open reading frame 118 (C6orf118) | 0.0285714 | 0.0161697 | 3.50181 |
| 115948 | Hypothetical protein (MGC20983) | 0.0285714 | 0.0388922 | 3.50146 |
| 4690 | NCK adaptor protein 1 (NCK1). | 0.0285714 | 0.00336022 | 3.47968 |
| 757 | Chromosome 21 open reading frame 4 (C21orf4) | 0.0285714 | 0.00572578 | 3.47953 |
| 8675 | Syntaxin 16 (STX16) | 0.0285714 | 0.0272295 | 3.47096 |
| 340419 | Hypothetical protein (MGC35555) | 0.0285714 | 0.0118447 | 3.44753 |
| 29915 | Host cell factor 2 | 0.0285714 | 0.00168338 | 3.44432 |
| 79640 | Hypothetical protein (FLJ23584) | 0.0285714 | 0.00729159 | 3.43226 |
| 57218 | KIAA1155 protein | 0.0285714 | 0.0492304 | 3.42522 |
| 54510 | Protocadherin 18 | 0.0285714 | 0.0289944 | 3.40734 |
| 166979 | CDC20-like protein | 0.0285714 | 0.0481074 | 3.39661 |
| 27300 | Zinc finger protein (AF020591) | 0.0285714 | 0.0105213 | 3.37317 |
| 63908 | Beta-soluble NSF attachment protein (Snap-Beta) [Source:SWISSPROT;Acc:Q9H115]" | 0.0285714 | 0.0222171 | 3.37296 |
| 55676 | Solute carrier family 30 (zinc transporter), member 6 | 0.0285714 | 0.0246941 | 3.36053 |
| 339448 | Hypothetical protein LOC339448 | 0.0285714 | 0.0213572 | 3.35582 |
| 80165 | Dynein, axonemal, heavy polypeptide 3 | 0.0285714 | 0.0253397 | 3.35213 |
| 55504 | Tumor necrosis factor receptor superfamily, member 19, transcript variant 1 | 0.0285714 | 0.00577939 | 3.34808 |
| 79964 | Hypothetical protein FLJ23529 | 0.0285714 | 0.0245308 | 3.32728 |
| 9073 | Claudin 8 | 0.0285714 | 0.0138762 | 3.32024 |
| 84293 | Hypothetical protein MGC4248 | 0.0285714 | 0.0185526 | 3.31946 |
| 284004 | Hypothetical protein (FLJ23825) | 0.0285714 | 0.0488853 | 3.30935 |
| 84102 | Hypothetical protein DKFZp434K0427 | 0.0285714 | 0.0151679 | 3.25386 |
| 90233 | Zinc finger protein 551 | 0.0285714 | 0.00937807 | 3.23261 |
| 54700 | RNA polymerase I transcription factor RRN3 | 0.0285714 | 0.0117821 | 3.2306 |
| 7586 | Zinc finger protein 36 (KOX 18) | 0.0285714 | 0.000900586 | 3.16544 |
| 8935 | Src family associated phosphoprotein 2 | 0.0285714 | 0.00780084 | 3.16544 |
| 54839 | NM_017691:hypothetical protein FLJ20156 (FLJ20156), mRNA. | 0.0285714 | 0.0299775 | 3.1424 |
| 83872 | Hemicentin (FIBL-6) | 0.0285714 | 0.0295906 | 3.1384 |
| 28994 | PRO0471 protein | 0.0285714 | 0.00025226 | 3.13406 |
| 114880 | Oxysterol binding protein-like 6 | 0.0285714 | 0.00366424 | 3.1251 |
| 64968 | Mitochondrial ribosomal protein S6 | 0.0285714 | 0.0175914 | 3.10662 |
| 84142 | Hypothetical protein (FLJ13614) | 0.0285714 | 0.0470934 | 3.09808 |
| 54503 | HIP14-related protein (HIP14L) | 0.0285714 | 0.0128155 | 3.09268 |
| 80209 | Hypothetical protein (FLJ12661) | 0.0285714 | 0.0354873 | 3.09218 |
| 9946 | Crystallin, zeta (quinone reductase)-like, transcript variant 3 | 0.0285714 | 0.0244315 | 3.0919 |
| 115209 | Metalloprotease related protein 1 [Source:RefSeq;Acc:NM_145243] | 0.0285714 | 0.0302134 | 3.09186 |
| 125113 | Truncated type I keratin KA21 | 0.0285714 | 0.0172151 | 3.06109 |
| 5671 | Pregnancy specific beta-1-glycoprotein 3 (PSG3) | 0.0285714 | 0.00356771 | 3.05721 |
| 83894 | NYD-SP14 protein | 0.0285714 | 0.0473228 | 3.01779 |
| 79772 | Hypothetical protein (FLJ22344) | 0.0285714 | 0.0027062 | 2.99541 |
| 2256 | Fibroblast growth factor 11 (FGF11) | 0.0285714 | 0.0103558 | 2.9812 |
| 79623 | UDP-N-acetyl-alpha-D-galactosamine:polypeptide N-acetylgalactosaminyltransferase 14 | 0.0285714 | 0.0245616 | 2.97869 |
| 254778 | Hypothetical protein (MGC33510) | 0.0285714 | 0.025155 | 2.9737 |
| 345667 | Similar to ADAMTS-10 precursor (A disintegrin and (ADAM-TS10) | 0.0285714 | 0.0416996 | 2.93848 |
| 4246 | Secretoglobin, family 2A, member 1 | 0.0285714 | 0.00197668 | 2.93429 |
| 81626 | Chromosome 1 open reading frame 14 | 0.0285714 | 0.000256863 | 2.93016 |
| 64083 | Golgi phosphoprotein 3 (coat-protein) | 0.0285714 | 0.0118718 | 2.90758 |
| 84841 | Hypothetical protein MGC15634 | 0.0285714 | 0.0220479 | 2.86965 |
| 5494 | Protein phosphatase 1A (formerly 2C), magnesium-dependent, alpha isoform" | 0.0285714 | 0.0333916 | 2.86748 |
| 80013 | Hypothetical protein (FLJ13397) | 0.0285714 | 0.0211579 | 2.85573 |
| 57504 | Metastasis associated family, member 3 | 0.0285714 | 0.0474754 | 2.85103 |
| 85460 | KIAA1729 protein | 0.0285714 | 0.0406882 | 2.80274 |
| 79971 | Chromosome 1 open reading frame 139 | 0.0285714 | 0.0119946 | 2.7998 |
| 9202 | Zinc finger protein 262 | 0.0285714 | 0.0420672 | 2.78214 |
| 2117 | Ets variant gene 3 (ETV3) | 0.0285714 | 0.00459737 | 2.75904 |
| 22897 | Centrosomal protein 164kDa | 0.0285714 | 0.0184448 | 2.75478 |
| 3209 | Homeo box A13 (HOXA13). | 0.0285714 | 0.00180275 | 2.74858 |
| 9525 | Vacuolar protein sorting 4B (yeast) | 0.0285714 | 0.00428317 | 2.73416 |
| 79760 | Gem (nuclear organelle) associated protein 7 | 0.0285714 | 0.0342612 | 2.72294 |
| 66004 | Ly-6 neurotoxin-like protein 1 (LYNX1), transcript variant 1 | 0.0285714 | 0.0385452 | 2.71654 |
| 11337 | GABA(A) receptor-associated protein | 0.0285714 | 0.0256542 | 2.71508 |
| 84688 | Chromosome 9 open reading frame 24 (C9orf24), transcript variant 2 | 0.0285714 | 0.0450399 | 2.66742 |
| 9627 | Synuclein, alpha interacting protein (synphilin) | 0.0285714 | 0.0452377 | 2.66574 |
| 157869 | RPE-spondin (RPESP) | 0.0285714 | 0.0466259 | 2.66263 |
| 170392 | Oncoprotein induced transcript 3 | 0.0285714 | 0.0169329 | 2.66182 |
| 26505 | Cyclin M3 | 0.0285714 | 0.0221511 | 2.63406 |
| 65268 | Protein kinase, lysine deficient 2 (PRKWNK2) | 0.0285714 | 0.0318297 | 2.60762 |
| 5336 | Phospholipase C, gamma 2 (phosphatidylinositol-specific) | 0.0285714 | 0.0401611 | 2.59452 |
| 7752 | Zinc finger protein 200 | 0.0285714 | 0.0418162 | 2.57264 |
| 3626 | Inhibin, beta C | 0.0285714 | 0.0195444 | 2.55419 |
| 154313 | Chromosome 6 open reading frame 165 | 0.0285714 | 0.004044 | 2.54051 |
| 54980 | Hypothetical protein (FLJ20558) | 0.0285714 | 0.0452131 | 2.53638 |
| 51067 | CGI-04 protein (CGI-04). | 0.0285714 | 0.0433202 | 2.49697 |
| 124152 | Hypothetical protein (MGC35048) | 0.0285714 | 0.0144703 | 2.48956 |
| 55030 | F-box only protein 34 (FBXO34) | 0.0285714 | 0.0394483 | 2.43794 |
| 122945 | Hypothetical protein (LOC122945) | 0.0285714 | 0.00582958 | 2.42524 |
| 23682 | RAB38, member RAS oncogene family | 0.0285714 | 0.0365978 | 2.37039 |
| 1362 | Carboxypeptidase D | 0.0285714 | 0.00543253 | 2.36742 |
| 192669 | Eukaryotic translation initiation factor 2C, 3 (EIF2C3), transcript variant 1 | 0.0285714 | 0.0392092 | 2.35671 |
| 3290 | Hydroxysteroid (11-beta) dehydrogenase 1 transcript variant 1 | 0.0285714 | 0.0181989 | 2.34891 |
| 253827 | Methionine sulfoxide reductase B3 | 0.0285714 | 0.0393142 | 2.32301 |
| 202451 | Hypothetical protein LOC202451 | 0.0285714 | 0.0286116 | 2.2118 |
| 121227 | Hypothetical protein (FLJ90440) | 0.0285714 | 0.0496336 | 2.18415 |
| 23563 | Carbohydrate (N-acetylglucosamine 6-O) sulfotransferase 5 | 0.0285714 | 0.0194974 | 2.15914 |
| 127254 | Hypothetical protein DKFZp547I048 | 0.0285714 | 0.0231653 | 2.14478 |
| 127733 | UBX domain containing 3 | 0.0285714 | 0.0188625 | 2.1347 |
| 905 | Cyclin T2 | 0.0285714 | 0.0397064 | 2.11994 |
| 29855 | Ubinuclein 1 (UBN1) | 0.0285714 | 0.00459397 | 2.11435 |
| 199920 | Complement component 8, alpha polypeptide | 0.0285714 | 0.0140213 | 2.0709 |
| 10085 | EGF-like repeats and discoidin I-like domains 3 | 0.0285714 | 0.000640782 | 2.00802 |
| 133 | Adrenomedullin | 0.0285714 | 0.029358 | 1.98808 |
| 81029 | Wingless-type MMTV integration site family, member 5B (WNT5B). | 0.0285714 | 0.0144028 | 1.97684 |
| 284485 | Hypothetical protein FLJ36032 | 0.0285714 | 0.0475019 | 1.95541 |
| 6495 | Sine oculis homeobox homolog 1 (Drosophila) | 0.0285714 | 0.0100782 | 1.92234 |
| 29886 | Sorting nexin 8 (SNX8) | 0.0285714 | 0.0019114 | 1.91549 |
| 5396 | Paired related homeobox 1 | 0.0285714 | 0.021219 | 1.90103 |
| 1139 | Cholinergic receptor, nicotinic, alpha polypeptide 7 | 0.0285714 | 0.00698351 | 1.8969 |
| 132660 | Hypothetical protein DKFZp686L1814 | 0.0285714 | 0.0469397 | 1.88585 |
| 272 | Adenosine monophosphate deaminase (isoform E) | 0.0285714 | 0.00652612 | 1.8748 |
| 65975 | Serine/Threonine kinase 33. | 0.0285714 | 0.000956004 | 1.85025 |
| 133690 | Hypothetical protein (MGC26610) | 0.0285714 | 0.040655 | 1.84626 |
| 112950 | Mediator of RNA polymerase II transcription, subunit 8 homolog (yeast) | 0.0285714 | 0.0394132 | 1.84606 |
| 102 | A disintegrin and metalloproteinase domain 10 | 0.0285714 | 0.0243648 | 1.84347 |
| 6273 | S100 calcium binding protein A2 | 0.0285714 | 0.0356401 | 1.78724 |
| 23145 | KIAA0543 protein | 0.0285714 | 0.0176873 | 1.77477 |
| 54558 | Spermatogenesis associated 6 | 0.0285714 | 0.0210547 | 1.77468 |
| 160418 | Hypothetical protein FLJ90492 | 0.0285714 | 0.00258269 | 1.77 |
| 2956 | MutS homolog 6 (E. coli) (MSH6) | 0.0285714 | 0.00091899 | 1.75864 |
| 257062 | Hypothetical protein (MGC39581) | 0.0285714 | 0.000442459 | 1.75542 |
| 283518 | Potassium channel regulator (KCNRG) | 0.0285714 | 0.0200473 | 1.74226 |
| 120376 | Hypothetical protein LOC120376 | 0.0285714 | 0.0485862 | 1.71393 |
| 376940 | FLJ41410 protein | 0.0285714 | 0.00471248 | 1.67813 |
| 23650 | Tripartite motif-containing 29 | 0.0285714 | 0.0360782 | 1.66814 |
| 222256 | Hypothetical protein (FLJ23834) | 0.0285714 | 0.00289215 | 1.65924 |
| 135138 | PARK2 co-regulated (PACRG) | 0.0285714 | 0.00811577 | 1.62416 |
| 51668 | HSPCO34 protein (LOC51668) | 0.0285714 | 0.0305569 | 1.61793 |
| 4753 | NEL-like 2 (chicken) | 0.0285714 | 0.0434669 | 1.60864 |
| 10126 | Dynein, axonemal, light polypeptide 4 | 0.0285714 | 0.0346145 | 1.60645 |
| 6657 | SRY (sex determining region Y)-box 2 (SOX2). | 0.0285714 | 0.0465066 | 1.5946 |
| 23007 | Phospholipase C-like 3 | 0.0285714 | 0.0177852 | 1.5785 |
| 128344 | Hypothetical protein (LOC128344) | 0.0285714 | 0.0304 | 1.57303 |
| 83853 | AKAP-associated sperm protein | 0.0285714 | 0.00789411 | 1.5712 |
| 79689 | Likely ortholog of mouse tumor necrosis-alpha-induced adipose-related protein | 0.0285714 | 0.0456712 | 1.54875 |
| 891 | Cyclin B1 | 0.0285714 | 0.0112727 | 1.54852 |
| 79645 | Hypothetical protein (FLJ11767) | 0.0285714 | 0.0238141 | 1.53795 |
| 57819 | LSM2 homolog, U6 small nuclear RNA associated (S. cerevisiae) | 0.0285714 | 0.0262391 | 1.5328 |
| 8649 | Mitogen-activated protein kinase kinase 1 interacting protein 1 | 0.0285714 | 0.00446736 | 1.52733 |
| 80736 | Chromosome 6 open reading frame 29 | 0.0285714 | 0.0190598 | 1.52598 |
| 7982 | Suppression of tumorigenicity 7 (ST7), transcript variant a | 0.0285714 | 0.00708451 | 1.52325 |
| 219621 | Hypothetical protein (MGC44593) | 0.0285714 | 0.0380722 | 1.50712 |
| 26064 | Retinoic acid induced 14 | 0.0285714 | 0.0224371 | 1.50548 |
| 254956 | Chromosome 9 open reading frame 18 | 0.0285714 | 0.020929 | 1.50257 |
| 57699 | Copine V (CPNE5) | 0.0285714 | 0.0103581 | 1.48718 |
| 63931 | Mitochondrial ribosomal protein S14 | 0.0285714 | 0.0243214 | 1.4731 |
| 6542 | Solute carrier family 7 (cationic amino acid transporter, y+ system), member 2" | 0.0285714 | 0.000162518 | 1.46916 |
| 23273 | KIAA0367 protein (BNIP2 motif containing molecule at the carboxyl terminal region 1) | 0.0285714 | 0.0242382 | 1.46348 |
| 5350 | Serologically defined breast cancer antigen NY-BR-15 . [Source:SPTREMBL;Acc:Q9H289] | 0.0285714 | 0.00297624 | 1.44859 |
| 80227 | Hypothetical protein (FLJ11848) | 0.0285714 | 0.0440343 | 1.40729 |
| 80206 | Formin homology 2 domain containing 3 | 0.0285714 | 0.0167256 | 1.40715 |
| 230 | Aldolase C, fructose-bisphosphate | 0.0285714 | 0.0194491 | 1.40415 |
| 8537 | Breast carcinoma amplified sequence 1 (BCAS1) | 0.0285714 | 0.00150423 | 1.40242 |
| 79740 | Hypothetical protein (FLJ23049) | 0.0285714 | 0.0247161 | 1.39315 |
| 26249 | Kelch-like 3 (Drosophila) (KLHL3) | 0.0285714 | 0.00525978 | 1.39019 |
| 89765 | Testes specific A2 homolog (mouse) (TSGA2) | 0.0285714 | 0.0206683 | 1.38524 |
| 51715 | RAB23, member RAS oncogene family" | 0.0285714 | 0.0301801 | 1.37755 |
| 84190 | Hypothetical protein (FLJ22789) | 0.0285714 | 0.0322119 | 1.36219 |
| 79898 | Hypothetical protein (FLJ13590). | 0.0285714 | 0.0299482 | 1.35569 |
| 220136 | Hypothetical protein (FLJ32743) | 0.0285714 | 0.01705 | 1.35182 |
| 149483 | Hypothetical protein (FLJ33084) | 0.0285714 | 0.0105226 | 1.34194 |
| 4974 | Neurofibromin 1 | 0.0285714 | 0.00368411 | 1.34087 |
| 11224 | Ribosomal protein L35 | 0.0285714 | 0.0219611 | 1.33381 |
| 84520 | Chromosome 14 open reading frame 142 (C14orf142) | 0.0285714 | 0.0272982 | 1.33028 |
| 196385 | Dynein, axonemal, heavy polypeptide 10 | 0.0285714 | 0.0435896 | 1.32499 |
| 55000 | Hypothetical protein FLJ20618 | 0.0285714 | 0.0081232 | 1.29925 |
| 84058 | Hypothetical protein FLJ12953 similar to Mus musculus D3Mm3e (FLJ12953) | 0.0285714 | 0.021979 | 1.29097 |
| 378464 | Protein containing single MORN motif in testis | 0.0285714 | 8.03E-05 | 1.27809 |
| 158798 | A-kinase anchoring protein 28 | 0.0285714 | 0.0377543 | 1.27388 |
| 128344 | Hypothetical protein (LOC128344) | 0.0285714 | 0.0209897 | 1.27063 |
| 8668 | Eukaryotic translation initiation factor 3, subunit 2 beta | 0.0285714 | 0.00195132 | 1.25425 |
| 9213 | Xenotropic and polytropic retrovirus receptor | 0.0285714 | 0.0369011 | 1.24911 |
| 126823 | Hypothetical protein (MGC33338), | 0.0285714 | 0.00243615 | 1.24558 |
| 222663 | Signal peptide, CUB domain, EGF-like 3 | 0.0285714 | 0.00639458 | 1.24269 |
| 6450 | SH3 domain binding glutamic acid-rich protein (SH3BGR). | 0.0285714 | 0.0154382 | 1.23619 |
| 79841 | Hypothetical protein (FLJ23598) | 0.0285714 | 0.000845855 | 1.23425 |
| 667 | Bullous pemphigoid antigen 1, (BPAG1) | 0.0285714 | 0.0173852 | 1.22752 |
| 2770 | Guanine nucleotide binding protein (G protein), alpha inhibiting activity polypeptide 1 (GNAI1) | 0.0285714 | 0.0152976 | 1.20647 |
| 92737 | Delta-notch-like EGF repeat-containing transmembrane | 0.0285714 | 0.0118928 | 1.20405 |
| 154796 | Angiomotin (AMOT). | 0.0285714 | 0.0375142 | 1.19599 |
| 11005 | Serine protease inhibitor, Kazal type, 5 (SPINK5), | 0.0285714 | 0.0110372 | 1.18824 |
| 150696 | Prominin 2 | 0.0285714 | 0.0279928 | 1.14851 |
| 124975 | Hypothetical protein (FLJ90165) | 0.0285714 | 0.0106314 | 1.14382 |
| 5494 | Protein phosphatase 1A (formerly 2C), magnesium-dependent, alpha isoform , transcript variant 3 | 0.0285714 | 0.0202692 | 1.13927 |
| 10103 | Tetraspan 1 (TSPAN-1) | 0.0285714 | 0.00344307 | 1.13728 |
| 3779 | Potassium large conductance calcium-activated channel, subfamily M, beta member 1 | 0.0285714 | 0.0109482 | 1.12549 |
| 23243 | Ankyrin repeat domain 28 | 0.0285714 | 0.00821055 | 1.12264 |
| 10239 | Adaptor-related protein complex 3, sigma 2 subunit | 0.0285714 | 0.00380844 | 1.11944 |
| 6240 | Ribonucleotide reductase M1 polypeptide | 0.0285714 | 0.0341047 | 1.11787 |
| 80854 | Histone-lysine N-methyltransferase, H3 Lysine-4 specific (EC 2.1.1.43) [Source:SWISSPROT;Acc:Q8WTS6]" | 0.0285714 | 0.0082644 | 1.11726 |
| 1399 | CRK-Like Protein. [Source:SWISSPROT;Acc:P46109] | 0.0285714 | 0.0238865 | 1.10894 |
| 973 | CD79A antigen (immunoglobulin-associated alpha) (CD79A) | 0.0285714 | 0.00726391 | 1.10481 |
| 5609 | Mitogen-activated protein kinase kinase 7 | 0.0285714 | 0.00181033 | 1.10077 |
| 7696 | Zinc finger protein 137 (clone pHZ-30) (ZNF137) | 0.0285714 | 0.00493685 | 1.09646 |
| 57728 | Repeat membrane protein PWDMP | 0.0285714 | 0.000238918 | 1.08765 |
| 51704 | G protein-coupled receptor, family C, group 5, member B | 0.0285714 | 0.000665636 | 1.07756 |
| 8382 | Non-metastatic cells 5, protein expressed in (nucleoside-diphosphate kinase) (NME5). | 0.0285714 | 0.000137988 | 1.07385 |
| 5179 | Proenkephalin | 0.0285714 | 0.025538 | 1.06976 |
| 132671 | SPATA18 spermatogenesis associated 18 homolog (rat) | 0.0285714 | 0.0409465 | 1.0581 |
| 59084 | Ectonucleotide pyrophosphatase/phosphodiesterase 5 | 0.0285714 | 0.0271826 | 1.05753 |
| 5239 | Phosphoglucomutase 5 (PGM5) | 0.0285714 | 0.0131923 | 1.05555 |
| 23090 | OLF-1/EBF associated zinc finger gene (OAZ) | 0.0285714 | 0.00226302 | 1.05223 |
| 4135 | Microtubule-associated protein 6 | 0.0285714 | 0.0266878 | 1.05158 |
| 284252 | Potassium channel tetramerisation domain containing 1 | 0.0285714 | 0.000115574 | 1.03779 |
| 118491 | Tetratricopeptide repeat-containing protein (LOC118491) | 0.0285714 | 0.0222426 | 1.03727 |
| 7991 | Putative prostate cancer tumor suppressor (N33). | 0.0285714 | 0.0079167 | 1.03514 |
| 90557 | Hypothetical protein BC016861 | 0.0285714 | 0.0191449 | 1.03294 |
| 10451 | Vav 3 oncogene (VAV3) | 0.0285714 | 0.0426688 | 1.03248 |
| 930 | CD19 antigen | 0.0285714 | 0.0259134 | 1.03034 |
| 114827 | Forkhead-associated (FHA) phosphopeptide binding domain 1 | 0.0285714 | 0.00472139 | 1.02814 |
| 63920 | Transposon-derived Buster3 transposase-like | 0.0285714 | 0.0151578 | 1.02039 |
| 27239 | Likely ortholog of mouse gene rich cluster, A gene, transcript variant A-2. | 0.0285714 | 0.0126233 | 1.01702 |
| 80129 | Chromosome 6 open reading frame 97 | 0.0285714 | 0.00933623 | 1.01578 |
| 7049 | Transforming growth factor, beta receptor III (betaglycan) | 0.0285714 | 0.0194547 | 1.01335 |
| 260293 | Likely ortholog of rat cytochrome P450 4X1 (CYP4X1) | 0.0285714 | 0.00120393 | 1.00137 |
