# Supplementary material for: Accelerated Variant of Idiopathic Pulmonary Fibrosis: Clinical Behavior and Gene Expression Pattern
Source: PLoS One. 2007 May 30;2(5):e482. doi: 10.1371/journal.pone.0000482 (PMC1868965; doi:10.1371/journal.pone.0000482)
Supplement: Table S2 — Downregulated Genes in Rapid Progressors (0.16 MB DOC) [file pone.0000482.s002.doc]

## Table S2

**Downregulated Genes in Rapid Progressors**

| **GenBank Accn Locus Link** | **Gene** | **TNOM PValue** | **t-Test PValue** | **Fold Change**  **(log base 2)** |
| --- | --- | --- | --- | --- |
| 9173 | Interleukin 1 receptor-like 1 | 0.0285714 | 0.00112714 | -6.24996 |
| 84892 | Hypothetical protein (FLJ14566) | 0.0285714 | 1.29E-07 | -5.73785 |
| 9022 | Chloride intracellular channel 3 (CLIC3) | 0.0285714 | 5.92E-05 | -5.67 |
| 2974 | Guanylate cyclase 1, soluble, beta 2 | 0.0285714 | 2.12E-06 | -4.89978 |
| 80829 | Ciliary neurotrophic factor (CNTF) | 0.0285714 | 3.30E-05 | -4.8525 |
| 91289 | Hypothetical protein BC002942 (BC002942) | 0.0285714 | 0.00115994 | -4.77363 |
| 151126 | Hypothetical protein (FLJ25270), | 0.0285714 | 5.10E-06 | -4.74622 |
| 89882 | Protein kinase NYD-SP25 | 0.0285714 | 0.000686623 | -4.73714 |
| 5079 | Paired box gene 5 (B-cell lineage specific activator protein) (PAX5) | 0.0285714 | 8.06E-06 | -4.6577 |
| 166929 | Hypothetical protein (MGC26963) | 0.0285714 | 0.00213517 | -4.57936 |
| 6532 | Solute carrier family 6 (neurotransmitter transporter, serotonin), member 4 (SLC6A4) | 0.0285714 | 0.0307418 | -4.54655 |
| 6921 | Transcription elongation factor B (SIII), polypeptide 1 (15kDa, elongin C) (TCEB1) | 0.0285714 | 0.00545405 | -4.5098 |
| 55331 | Phytoceramidase, alkaline (PHCA) | 0.0285714 | 0.000853898 | -4.47832 |
| 5134 | Programmed cell death 2 (PDCD2), transcript variant 1 | 0.0285714 | 1.08E-05 | -4.46038 |
| 154214 | IBR domain containing 1 (IBRDC1) | 0.0285714 | 0.00219206 | -4.45462 |
| 79674 | Hypothetical protein (FLJ12604) | 0.0285714 | 0.00347781 | -4.43366 |
| 285682 | Fms-related tyrosine kinase 4 | 0.0285714 | 0.010834 | -4.42684 |
| 135656 | Diffuse panbronchiolitis critical region (DPCR1) | 0.0285714 | 0.00081253 | -4.42248 |
| 762 | Carbonic anhydrase IV (CA4) | 0.0285714 | 0.0122714 | -4.42053 |
| 8539 | Apoptosis inhibitor 5 (API5) | 0.0285714 | 3.36E-06 | -4.41638 |
| 55915 | LanC lantibiotic synthetase component C-like 2 (bacterial) (LANCL2) | 0.0285714 | 0.000746851 | -4.3659 |
| 56269 | Hypothetical protein R30953_1 (R30953_1) | 0.0285714 | 0.00186554 | -4.36132 |
| 7273 | Titin (TTN), transcript variant N2-B | 0.0285714 | 0.00365412 | -4.32474 |
| 6713 | Squalene epoxidase | 0.0285714 | 0.018322 | -4.31577 |
| 8085 | Protein kinase, AMP-activated, gamma 1 non-catalytic subunit (PRKAG1) | 0.0285714 | 8.93E-05 | -4.28871 |
| 91179 | Scavenger receptor class F, member 2, transcript variant 1 | 0.0285714 | 0.00103601 | -4.22654 |
| 64852 | Hypothetical protein FLJ22347 | 0.0285714 | 0.000424939 | -4.20882 |
| 85300 | Ataxia, cerebellar, Cayman type (caytaxin) | 0.0285714 | 0.000398963 | -4.20534 |
| 55009 | Hypothetical protein (FLJ20640) | 0.0285714 | 0.0032739 | -4.20438 |
| 1982 | Eukaryotic translation initiation factor 4 gamma, 2 | 0.0285714 | 0.000351921 | -4.1344 |
| 5449 | POU domain, class 1, transcription factor 1 (Pit1, growth hormone factor 1) | 0.0285714 | 0.00300838 | -4.10098 |
| 2868 | G protein-coupled receptor kinase 2-like (Drosophila) | 0.0285714 | 0.00735436 | -4.0863 |
| 84915 | Hypothetical protein (FLJ14721), | 0.0285714 | 0.000564631 | -4.07641 |
| 1191 | Clusterin | 0.0285714 | 1.16E-09 | -4.06168 |
| 55610 | Hypothetical protein LOC55610 | 0.0285714 | 0.00213582 | -4.01464 |
| 140612 | Zinc finger protein 28 homolog (mouse) (ZFP28) | 0.0285714 | 0.00107387 | -4.00684 |
| 54918 | Chemokine-like factor superfamily 6. [Source:RefSeq;Acc:NM_017801] | 0.0285714 | 0.00147143 | -4.00581 |
| 91754 | NIMA (never in mitosis gene a)- related kinase 9 (NEK9) | 0.0285714 | 0.00105437 | -3.96454 |
| 57715 | Sema domain, immunoglobulin domain (Ig), transmembrane | 0.0285714 | 0.00862933 | -3.94386 |
| 84918 | Low density lipoprotein receptor-related protein 11 | 0.0285714 | 0.000892347 | -3.94264 |
| 79628 | SH3 domain and tetratricopeptide repeats 2 | 0.0285714 | 0.000404373 | -3.91656 |
| 79923 | Nanog homeobox (NANOG) | 0.0285714 | 0.00847238 | -3.91065 |
| 63977 | PR domain containing 15 | 0.0285714 | 0.00198034 | -3.83952 |
| 219790 | Rhotekin 2 (RTKN2) | 0.0285714 | 0.00434028 | -3.83304 |
| 151556 | G protein-coupled receptor 155 | 0.0285714 | 0.00978328 | -3.81615 |
| 9142 | Chromosome X open reading frame 1 (CXorf1) | 0.0285714 | 2.58E-05 | -3.7868 |
| 79968 | Hypothetical protein (FLJ12973), | 0.0285714 | 0.00368177 | -3.77582 |
| 9702 | Translokin (KIAA0092) | 0.0285714 | 0.00940642 | -3.73258 |
| 8844 | Kinase suppressor of ras | 0.0285714 | 0.0068325 | -3.7252 |
| 7364 | UDP glycosyltransferase 2 family, polypeptide B7 (UGT2B7) | 0.0285714 | 0.00766486 | -3.71436 |
| 161145 | Hypothetical protein FLJ33387 | 0.0285714 | 0.00803792 | -3.67032 |
| 79608 | Tubby homolog (mouse) (TUB), transcript variant 1 | 0.0285714 | 0.0141922 | -3.62633 |
| 10017 | BCL2-like 10 (apoptosis facilitator) (BCL2L10) | 0.0285714 | 0.0062929 | -3.614 |
| 9388 | Lipase, endothelial (LIPG) | 0.0285714 | 0.0267816 | -3.5749 |
| 10568 | Solute carrier family 34 (sodium phosphate), member 2 | 0.0285714 | 0.0016321 | -3.54207 |
| 745 | Chromosome 11 open reading frame 9 (C11orf9) | 0.0285714 | 0.0311644 | -3.52024 |
| 84076 | Hypothetical protein (DKFZP434L1717) | 0.0285714 | 0.00105399 | -3.51008 |
| 10992 | Splicing factor 3b, subunit 2, 145kDa | 0.0285714 | 0.0114558 | -3.49625 |
| 3249 | Hepsin (transmembrane protease, serine 1) (HPN), transcript variant 2 | 0.0285714 | 0.0408055 | -3.48495 |
| 9735 | Kinetochore associated 1 (KNTC1) | 0.0285714 | 0.00440701 | -3.45968 |
| 5861 | RAB1A, member RAS oncogene family | 0.0285714 | 0.00068222 | -3.43621 |
| 2898 | Glutamate receptor, ionotropic, kainate 2 (GRIK2), transcript variant 1 | 0.0285714 | 0.00476755 | -3.436 |
| 10693 | Chaperonin containing TCP1, subunit 6B (zeta 2) (CCT6B) | 0.0285714 | 0.0110475 | -3.42558 |
| 200894 | Hypothetical protein DKFZp761H079 | 0.0285714 | 0.00295625 | -3.39615 |
| 84514 | D11lgp1e-like (LGP1) | 0.0285714 | 0.0038749 | -3.39056 |
| 105 | Adenosine deaminase, RNA-specific, B2 (RED2 homolog rat) (ADARB2) | 0.0285714 | 0.00161806 | -3.38804 |
| 115950 | Hypothetical protein BC016816 (LOC115950) | 0.0285714 | 0.0118254 | -3.37834 |
| 84807 | T-cell activation NFKB-like protein (TA-NFKBH) | 0.0285714 | 0.0358466 | -3.35086 |
| 85406 | DnaJ protein (DNAJ) | 0.0285714 | 0.00176921 | -3.34382 |
| 54474 | Keratin 20 (KRT20) | 0.0285714 | 0.00133961 | -3.34056 |
| 283225 | Hypothetical protein (FLJ37266) | 0.0285714 | 0.00551214 | -3.3381 |
| 64324 | Nuclear receptor binding SET domain protein 1 (NSD1), transcript variant 2 | 0.0285714 | 0.0037641 | -3.33409 |
| 197407 | Hypothetical protein (FLJ31751), | 0.0285714 | 0.0136968 | -3.29381 |
| 55544 | RNA-binding region (RNP1, RRM) containing 1 (RNPC1), transcript variant 1 | 0.0285714 | 0.0260079 | -3.24888 |
| 11173 | A disintegrin-like and metalloprotease (reprolysin type) with thrombospondin type 1 motif, 7 (ADAMTS7) | 0.0285714 | 0.00306704 | -3.20278 |
| 55824 | Phosphoprotein associated with glycosphingolipid-enriched microdomains | 0.0285714 | 0.0356909 | -3.19319 |
| 6161 | Ribosomal protein L32 | 0.0285714 | 0.0118303 | -3.15683 |
| 374618 | Testis expressed sequence 9 | 0.0285714 | 0.028234 | -3.08536 |
| 144097 | Hypothetical protein BC007540 | 0.0285714 | 0.0382414 | -3.05647 |
| 9231 | Discs, large (Drosophila) homolog 5 (DLG5) | 0.0285714 | 0.0318361 | -3.05643 |
| 79961 | Hypothetical protein (FLJ22457) | 0.0285714 | 0.00783276 | -2.98231 |
| 93587 | Hypothetical protein (MGC27034) | 0.0285714 | 0.0291245 | -2.89018 |
| 4091 | MAD, mothers against decapentaplegic homolog 6 (Drosophila) (MADH6) | 0.0285714 | 0.0127669 | -2.87009 |
| 79014 | Hypothetical protein (MGC3020) | 0.0285714 | 0.0139997 | -2.78363 |
| 90441 | Zinc finger-like protein 9 | 0.0285714 | 0.0124202 | -2.73601 |
| 51011 | CGI-105 protein (CGI-105) | 0.0285714 | 0.0104671 | -2.72672 |
| 5602 | Mitogen-activated protein kinase 10 | 0.0285714 | 0.0186649 | -2.72574 |
| 57716 | Periaxin (PRX) | 0.0285714 | 0.000321464 | -2.65821 |
| 57720 | G protein-coupled receptor 107 | 0.0285714 | 0.0402952 | -2.63079 |
| 10223 | Glycoprotein A33 (transmembrane) (GPA33), | 0.0285714 | 0.0477356 | -2.60316 |
| 79813 | Euchromatic histone methyltransferase 1 (Eu-HMTase1) | 0.0285714 | 0.00170752 | -2.4676 |
| 9992 | Potassium voltage-gated channel, Isk-related family, member 2 (KCNE2) | 0.0285714 | 0.0036981 | -2.44551 |
| 7433 | Vasoactive intestinal peptide receptor 1 | 0.0285714 | 0.00264404 | -2.36054 |
| 84330 | Hypothetical protein (MGC15716) | 0.0285714 | 0.0497058 | -2.33181 |
| 79727 | Lin-28 homolog (C. elegans) | 0.0285714 | 0.0316689 | -2.28362 |
| 9877 | Hs.17969:KIAA0663 gene product | 0.0285714 | 0.0443157 | -2.24593 |
| 84281 | Hypothetical protein (MGC13057), | 0.0285714 | 0.0057756 | -2.23066 |
| 56729 | Resistin (RETN) | 0.0285714 | 0.00485963 | -1.85473 |
| 3620 | Indoleamine-pyrrole 2,3 dioxygenase (INDO) | 0.0285714 | 0.00860897 | -1.78939 |
| 547 | Kinesin family member 1A (KIF1A) | 0.0285714 | 0.0223864 | -1.77532 |
| 284029 | Hypothetical protein FLJ34790 (FLJ34790) | 0.0285714 | 0.0253078 | -1.73583 |
| 80339 | Chromosome 22 open reading frame 20 (C22orf20) | 0.0285714 | 0.0400358 | -1.56908 |
| 3002 | Granzyme B (granzyme 2, cytotoxic T-lymphocyte-associated serine esterase 1) | 0.0285714 | 0.00556152 | -1.55484 |
| 55187 | Hypothetical protein FLJ10619 | 0.0285714 | 0.0298909 | -1.46499 |
| 4117 | Male germ cell-associated kinase (MAK) | 0.0285714 | 0.0281698 | -1.44727 |
| 84875 | Hypothetical protein FLJ14464 | 0.0285714 | 0.0291427 | -1.43417 |
| 177 | Advanced glycosylation end product-specific receptor (AGER), transcript variant 1 | 0.0285714 | 0.0209846 | -1.39263 |
| 744 | Chromosome 11 open reading frame 8 (C11orf8) | 0.0285714 | 0.00299769 | -1.3649 |
| 10663 | Chemokine (C-X-C motif) receptor 6 (CXCR6) | 0.0285714 | 0.00117639 | -1.36341 |
| 348162 | LOC348162 hypothetical protein 348162 | 0.0285714 | 0.0129815 | -1.31484 |
| 11254 | Solute carrier family 6 (neurotransmitter transporter), member 14 (SLC6A14) | 0.0285714 | 0.00231667 | -1.30388 |
| 6764 | Suppression of tumorigenicity 5 (ST5), transcript variant 1 | 0.0285714 | 0.0277141 | -1.29596 |
| 9806 | Sparc/osteonectin, cwcv and kazal-like domains proteoglycan (testican) 2 | 0.0285714 | 0.00313015 | -1.29468 |
| 3248 | Hydroxyprostaglandin dehydrogenase 15-(NAD) | 0.0285714 | 0.00486889 | -1.29188 |
| 10412 | TGF beta-inducible nuclear protein 1 | 0.0285714 | 0.0135309 | -1.2853 |
| 7114 | Thymosin, beta 4, X-linked (TMSB4X) | 0.0285714 | 0.011713 | -1.28365 |
| 84689 | Testes development-related NYD-SP21 | 0.0285714 | 8.69E-06 | -1.28122 |
| 6646 | Sterol O-acyltransferase (acyl-Coenzyme A: cholesterol acyltransferase) 1 | 0.0285714 | 0.00923637 | -1.28014 |
| 153 | Adrenergic, beta-1-, receptor | 0.0285714 | 0.0101308 | -1.19737 |
| 94 | Activin A receptor type II-like 1 (ACVRL1) | 0.0285714 | 0.00918193 | -1.0961 |
| 55117 | Homolog of rat orphan transporter v7-3 | 0.0285714 | 0.00871265 | -1.05867 |
| 55273 | Hypothetical protein (FLJ10970) | 0.0285714 | 0.0249548 | -1.02279 |
| 10458 | BAI1-associated protein 2 | 0.0285714 | 0.0205751 | -1.00682 |
